# *In Vitro* and *In Vivo* Anti-Schistosomal Activity of the Alkylphospholipid Analog Edelfosine

**DOI:** 10.1371/journal.pone.0109431

**Published:** 2014-10-10

**Authors:** Edward Yepes, Rubén E. Varela-M, Julio López-Abán, E. L. Habib Dakir, Faustino Mollinedo, Antonio Muro

**Affiliations:** 1 IBSAL-CIETUS (Instituto de Investigación Biomédica de Salamanca-Centro de Investigación de Enfermedades Tropicales de la Universidad de Salamanca), Facultad de Farmacia, Universidad de Salamanca, Salamanca, Spain; 2 Instituto de Biología Molecular y Celular del Cáncer, Centro de Investigación del Cáncer, CSIC-Universidad de Salamanca, Campus Miguel de Unamuno, Salamanca, Spain; Royal Tropical Institute, Netherlands

## Abstract

**Background:**

Schistosomiasis is a parasitic disease caused by trematodes of the genus *Schistosoma*. Five species of *Schistosoma* are known to infect humans, out of which *S. haematobium* is the most prevalent, causing the chronic parasitic disease schistosomiasis that still represents a major problem of public health in many regions of the world and especially in tropical areas, leading to serious manifestations and mortality in developing countries. Since the 1970s, praziquantel (PZQ) is the drug of choice for the treatment of schistosomiasis, but concerns about relying on a single drug to treat millions of people, and the potential appearance of drug resistance, make identification of alternative schistosomiasis chemotherapies a high priority. Alkylphospholipid analogs (APLs), together with their prototypic molecule edelfosine (EDLF), are a family of synthetic antineoplastic compounds that show additional pharmacological actions, including antiparasitic activities against several protozoan parasites.

**Methodology/Principal Findings:**

We found APLs ranked edelfosine> perifosine> erucylphosphocholine> miltefosine for their *in vitro* schistosomicidal activity against adult *S. mansoni* worms. Edelfosine accumulated mainly in the worm tegument, and led to tegumental alterations, membrane permeabilization, motility impairment, blockade of male-female pairing as well as induction of apoptosis-like processes in cells in the close vicinity to the tegument. Edelfosine oral treatment also showed *in vivo* schistosomicidal activity and decreased significantly the egg burden in the liver, a key event in schistosomiasis.

**Conclusions/Significance:**

Our data show that edelfosine is the most potent APL in killing *S. mansoni* adult worms *in vitro*. Edelfosine schistosomicidal activity seems to depend on its action on the tegumental structure, leading to tegumental damage, membrane permeabilization and apoptosis-like cell death. Oral administration of edelfosine diminished worm and egg burdens in *S. mansoni*-infected CD1 mice. Here we report that edelfosine showed promising antischistosomal properties *in vitro* and *in vivo*.

## Introduction

Schistosomiasis is a parasitic disease caused by trematodes of the genus *Schistosoma*, being a major problem of public health. The genus *Schistosoma* contains 21 species, which are classified into four groups according to the geographic distribution, morphology of the parasite's eggs, and the intermediate host. Five major schistosome species are able to infect humans: *S. haematobium*, *S. mansoni*, *S. intercalatum*, *S. japonicum*, and *S. mekongi*, although human infections by *S. malayensis*, *S. mattheei*, and *S. guineensis* have also been described. Schistosomiasis is acquired by contact with freshwater contaminated with cercariae larvae, which actively penetrate mammal skin and transform into the schistosomula phase, migrating toward the lungs and then re-entering the venous circulation [1]. Both male and female schistosome parasites achieve sexual maturity in the bloodstream, then sexual reproduction occurs with the deposition of hundreds to thousands of eggs per day. Deposition of *Schistosome* eggs in the tissues is a stimulus to the influx of immune cells that leads to the development of a granulomatous reaction. This immunological reaction protects the host by neutralizing the schistosome egg antigens and destroying eggs. However, the granulomas are the most important pathogenic event in schistosomiasis since the deposition of collagen and the development of fibrosis cause the fibro-obstructive disease. Nevertheless, paradoxically, the development of granulomatous inflammation around parasite eggs has an essential host-protective and facilitates the successful excretion of the eggs from the host [2]–[4]. The World Health Organization (WHO) announced in October 2001 that schistosomiasis epidemiology should be recalculated. It is estimated more than 700 million people in 78 countries endemic for schistosomiasis are at risk of this disease. Furthermore, 240 million people are infected (80% in sub-Saharan Africa), 120 million have symptoms, and 20 million have severe disease, which results in approximately 280,000 deaths annually [2], [5], [6]. An additional report even increases the estimate of infected people to 391–597 million [7]. Some authors report the significant impact of the morbidity caused by schistosomiasis, as reflected in the loss of 1.53 million disability-adjusted life years [1]. The disease is among the Neglected Tropical Diseases catalogued by the Global Plan to combat Neglected Tropical Diseases 2008–2015, and is considered to be the second most socioeconomically devastating parasitic disease by the WHO, immediately after malaria [8].

Praziquantel is the main drug for the treatment of schistosomiasis, since oxamniquine and metrifonate are not currently available [9], [10]. However, praziquantel does not prevent reinfection and its administration requires taking into consideration the stage of the disease. Praziquantel is highly efficient against adult worms, being less effective against juvenile parasites (7–35 days) [11]. In the chronic phase, the dose of praziquantel depends on the species involved [2], [12]. The fact that schistosomiasis treatment is limited to one single pharmaceutical presents the risk associated with the appearance of resistances. The WHO has recognized the necessity of identifying new compounds as alternatives to praziquantel. In the last decade, only derivatives of artemisinin have appeared as a complement to the therapy against schistosomiasis. The use of these derivatives in combination with praziquantel can be a good strategy of control, since the artemisinin derivatives are effective against the juvenile phases of the parasite [9]. In addition, the activity of the artemisinin derivatives as antiparasitic chemotherapy has been demonstrated in numerous clinical trials [13]–[15]. However, the use of these antimalarial drugs in the treatment and/or control of schistosomiasis might lead to the appearance of malaria resistance to these compounds. Several experts are warning about the putative emergence of resistance to praziquantel, due to its massive use in schistosomiasis control campaigns, and the existence of drug resistant parasites in laboratory isolates [16]. This makes it necessary to find new molecules or therapeutic targets for schistosomiasis control.

**Figure 1:**
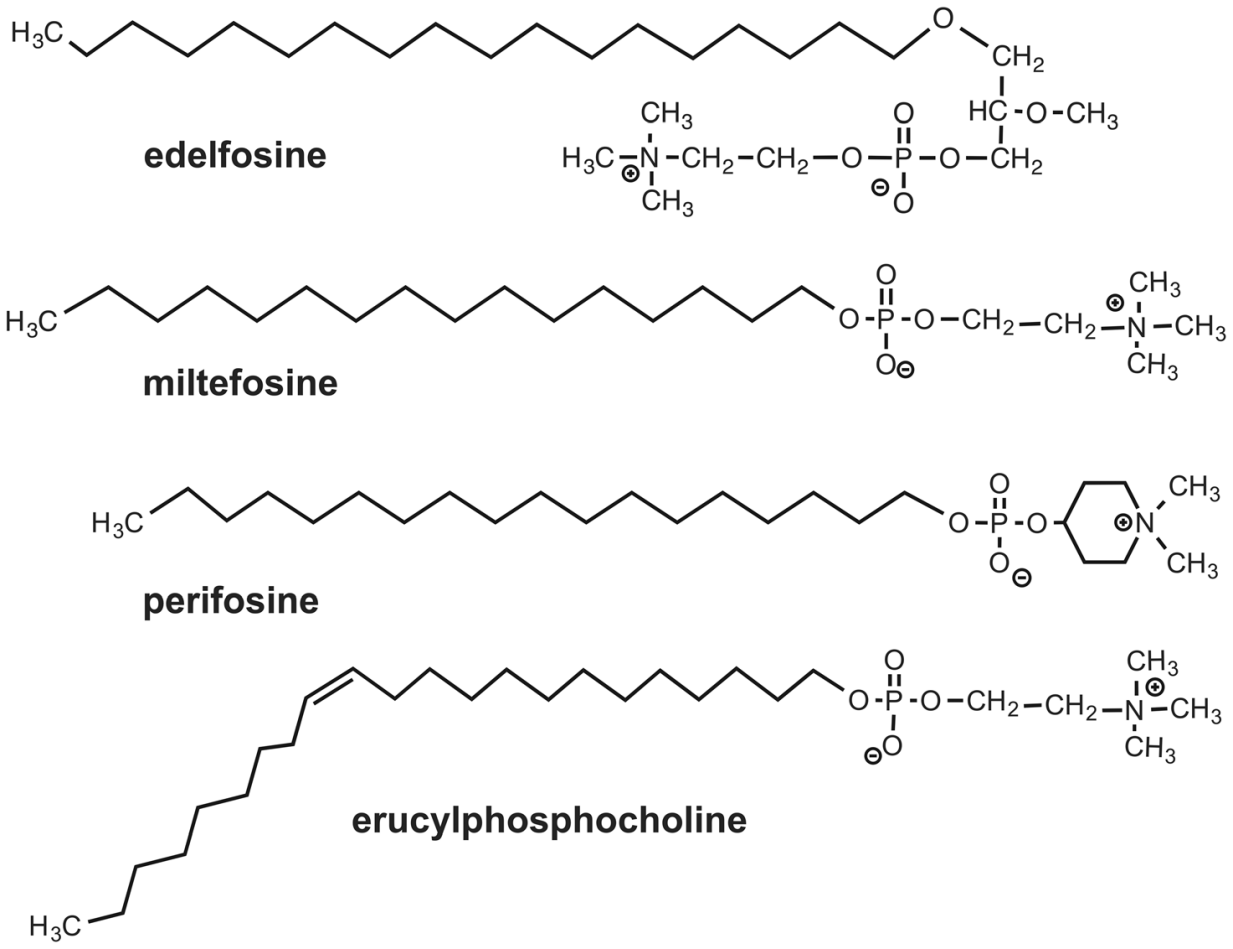
Chemical structures of edelfosine, miltefosine, perifosine and erucylphosphocholine.

Alkylphospholipid analogs (APLs) are a class of structurally related synthetic lipid compounds, including edelfosine, miltefosine, perifosine, and erucylphosphocholine (Figure 1), that act on cell membranes rather than on DNA [17]–[19]. Edelfosine (1-*O*-octadecyl-2-*O*-methyl-*rac*-glycero-3-phosphocholine), considered as the prototype APL molecule, is a promising antitumor ether phospholipid drug [20]–[22], which acts by activating apoptosis through its interaction with cell membranes [19], [23]–[25]. In addition to its antitumor activity, edelfosine has been shown to exert *in vitro* and *in vivo* antiparasitic activity against different species of *Leishmania* parasites [26]. Miltefosine has also been shown to exert activity against leishmaniasis and schistosomiasis [27], [28]. Both *Leishmania* and *Schistosoma* parasites share geographic areas in tropical and subtropical countries [29], [30]. Here we have investigated the putative anti-schistosomal properties of edelfosine, as compared to other APLs, by *in vitro* and *in vivo* approaches, using an experimental mouse model of *S. mansoni* infection.

## Materials and Methods

### Ethics statement

Animal procedures in this study complied with the Spanish (Ley 32/2007, Ley 6/2013 and Real Decreto 53/2013) and the European Union (European Directive 2010/63/EU) regulations on animal experimentation for the protection and humane use of laboratory animals, and were conducted at the accredited Animal Experimentation Facility of the University of Salamanca (Register number: PAE/SA/001). Procedures were approved by the Ethics Committee of the University of Salamanca (protocol approval number 48531). The animals' health status was monitored throughout the experiments by a health surveillance program according to Federation of European Laboratory Animal Science Associations (FELASA) guidelines.

### Drugs

Edelfosine (1-*O*-octadecyl-2-*O*-methyl-*rac*-glycero-3-phosphocoline) was obtained from R. Berchtold (Biochemisches Labor, Bern, Switzerland). Miltefosine (hexadecylphosphocholine) was from Calbiochem (Cambridge, MA). Perifosine (octadecyl-(1,1-dimethyl-piperidinio-4-yl)-phosphate) and erucylphosphocholine ((13Z)-docos-13-en-1-yl 2-(trimethylammonio)ethyl phosphate) were from Zentaris (Frankfurt, Germany). Stock sterile solutions of the distinct APLs (2 mM) were prepared in RPMI-1640 culture medium (Invitrogen, Carlsbad, CA), supplemented with 10% heat-inactivated fetal bovine serum (FBS), 2 mM glutamine, 100 IU/mL penicillin, and 100 µg/mL streptomycin, as previously described [20]. Praziquantel (PZQ) was obtained as Biltricide tablets (Bayer Vital, Leverkusen, Germany) and was dispersed in water with 2–2.5% Cremophor A6 oil-in-water emulsifier (Sigma, MO).

### Parasite maintenance, recovery and culture

Cercariae of *Schistosoma mansoni* (LE strain) were maintained routinely in *Biomphalaria glabrata* snails and female CD1 mice (Charles River, Criffa S.A., Barcelona, Spain) at University of Salamanca. Mice were maintained in polycarbonate and wire standard cages for 10 mice with food and water *ad libitum* in an environment under an alternating 12/12-h light-dark cycle at 20°C. Six-week-old females weighing 23–26 g were percutaneously infected with 150 *S. mansoni* cercariae per animal after being restrained with a mixture of ketamine 50 mg/kg of body weight, diazepam 5 mg/kg and atropine 1 mg/kg injected intraperitoneally. After 7–8 weeks, mice were killed with a lethal dose of 60 mg/kg of pentobarbital plus heparin (2 IU/mL) and then perfused aseptically with PBS and heparin (500 IU/L) to obtain couples, males and females from the portal and mesenteric veins [31], [32]. The worms were washed in RPMI-1640 culture medium (Invitrogen, Carlsbad, CA), kept at pH 7.5 with 20 mM HEPES and supplemented with 10% heat-inactivated fetal bovine serum (FBS), 2 mM L-glutamine, 100 IU/mL penicillin, and 100 µg/mL streptomycin, as previously described [31]. All mice were monitored daily by the staff from Animal Experimentation facility and after day 42 post-infection by a member of the research group qualified in animal welfare. Animals presenting any deterioration of the health status were killed with a lethal dose of pentobarbital 60 mg/kg.

### *In vitro* antischistosomal activity

Adult worms washed in RPMI-1640 were collected, counted, and transferred to RPMI-1640 medium (supplemented with 20 mM HEPES, 100 IU/mL penicillin, 100 µg/mL streptomycin and 2 mM L-glutamine). The worms were rinsed twice with this medium and distributed one pair of adult worms per well in 24-well culture plates. FBS was added to a final concentration of 10%, and the cultures were incubated at 37°C in a 5% CO_2_ atmosphere. After 2 h of culture to allow for adaptation, APLs were added to a final concentration of 1 to 80 µM (from a stock solution of 2 mM in RPMI-1640). The effects of APLs on *S. mansoni* adult worms were monitored every 24 h during 168 h for control groups and APL treatments. A number of parameters were evaluated to assess worm general condition, including: motility, alterations in the tegument, mortality rate, and changes in pairing [33]. The worms were considered dead when no movement was detected for at least 2 min of examination [34]. Additional criteria for viability were the MTT assay (see below), as well as pairing, egg production, and video-imaging to record morphological alterations and dead worms [35]. Worms were observed under a Nikon eclipse Ti inverted research microscope (Nikon Instruments, Amstelveen, The Netherlands). Video recordings were taken using a ProgRes C3 camera and a ProgRes MAC CapturePro software version 2.7 (JENOPTIK Optical Systems, Jena, Germany). As controls, pairs of *S. mansoni* adult worms were incubated in the presence RPMI-1640 (negative control) or were killed by heating at 56°C or treatment with 10 µM PZQ (positive control). All experiments were carried in quadruplicate.

### Assay for parasite viability

A three-step colorimetric quantitative assay based on 3-(4,5-dimethylthiazol-2-yl)-2,5-diphenyl tetrazolium bromide (MTT) was used to assess parasite viability [36]. The MTT assay is based on the uptake of the tetrazolium salt by viable/living cells followed by its reduction in the mitochondria to purple formazan, the colored product. The amount of formazan produced is proportional to the number of living cells, giving a measure of viability. In this study, worms were distributed (one pair of adult worms per well) in 96-well culture plates, containing 100 µL of phosphate buffered saline (PBS) with 0.5 mg MTT/mL for 30 min at 37°C. The solution was carefully removed and replaced with 200 µL dimethylsulfoxide (DMSO). Worms incubated in drug-free RPMI-1640 medium (negative control group) were allowed to stand in DMSO at room temperature for 1 h, and absorbance was read at 550 nm using an ELISA reader (Anthos 2010; Anthos Labtec Instruments, Wals, Austria). Heat-killed worms at 56°C and worms treated with 10 µM PZQ were used as positive control groups. APLs were added to a final concentration of 1 to 80 µM (from a stock solution of 2 mM in RPMI-1640) for up to 168 h incubation.

We studied the incorporation of propidium iodide (PI) in adult worms to assess the differential membrane permeability to this dye. The effect of edelfosine (1–80 µM) was analyzed following incubation for 24 h. After washing the parasites, PI was simultaneously added to each well of the microtiter plate to obtain a final concentration of 2.0 mg/mL in 96-well microtiter plates. Fluorescently labeled parasites were subsequently detected using a BioTek Synergy 2 (BioTek Instruments, Winooski, VT) fluorescent plate reader containing appropriate filters (485/20 nm excitation, 645/20 nm emission). All experiments were carried in quadruplicate.

### Distribution of fluorescent edelfosine analog in *S. mansoni*

Four couples of adult worms were transferred and maintained in RPMI-1640 medium supplemented with 20 mM HEPES, 100 IU/mL penicillin, 100 µg/mL streptomycin. Each well of a 24-well culture plate containing 2 mL of RPMI-1640 culture medium and two parasitic couples was incubated for 1 h with 10 µM of the fluorescent edelfosine 1-*O*-[11′-(6″-ethyl-1″,3″,5″,7″-tetramethyl-4″,4″-difluoro-4″-bora-3a″,4a″-diaza-s-indacen-2″-yl)undecyl]-2-*O*-methyl-*rac*-glycero-3-phosphocholine [37], [38]. In addition, two parasitic couples were incubated in RPMI-1640 only (control of autofluorescence). Parasites were analyzed with a Zeiss Axioplan 2 fluorescence microscope (Carl Zeiss, Oberkochen, Germany). All experiments were performed in quadruplicate.

### Assessment of apoptosis by TUNEL Assay

The DeadEnd Fluorometric TUNEL System (Promega, Madison, WI) was used to detect apoptosis. Parasite sections (4 µm) were deparaffinized in xylene, and rehydrated in graded ethanol and distilled water. After being rinsed three time with PBS, the slides were permeabilized with proteinase K (20 µg/mL) in proteinase K buffer (100 mM Tris-HCl, pH 8.0, 50 mM EDTA) for 15 min at room temperature. The slides were incubated with equilibrium buffer [200 mM potassium cacodylate, 25 mM Tris-HCl, pH 6.6, 0.2 mM dithiothreitol (DTT), 0.25 mg/mL bovine serum albumin, 2.5 mM cobalt chloride] at room temperature for 30 min, followed by assessment of cell apoptosis with a terminal deoxynucleotidyl transferase dUTP nick end labeling kit according to the manufacturer's instructions, and using fluorescence microscopy (Carl Zeiss). In histological sections, the apoptotic index, defined as the percentage of apoptotic cells, was used as a quantitative measure of apoptosis. The apoptotic index was determined as follows: (number of TUNEL-positive cells/total number of cells) x 100.

### Histopathological analysis

Parasites were fixed in 4% buffered paraformaldehyde and embedded in paraffin. The tissue sections of schistosomes (4 µm) were deparaffinized and hydrated in graded ethanol and PBS, and stained with hematoxylin and eosin (H&E). Histopathological evaluation was done under microscope analysis. The slides were viewed using an Olympus BX51 microscope coupled with Olympus DP Controller software to capture images [39], [40].

### *In vivo* study of antischistosomal activity

Six-week-old female CD1 mice (Charles River), weighing 23–26 g, were kept in the Animal Experimentation Facility. Mice were randomly allocated into three experimental groups and all were chemically restrained and percutaneously exposed to 150 *S. mansoni* cercariae per animal [32]. Group 1 received the vehicle solution (water) via oral administration from day 42 to day 52 post-infection (p.i.) (n = 7); group 2 were orally treated with 500 mg/kg/day of PZQ on days 47 and 48 p.i. (n = 7); and group 3 were orally treated with 45 mg/kg/day of edelfosine from day 42 and 52 p.i. (n = 7). Mice were euthanized at week 9 p.i. with a lethal dose of pentobarbital (60 mg/kg), and then perfused to recover adult worms from portal and mesenteric veins [31], [32]. The number of parasite eggs per gram (epg) in liver was counted after digestion with 25 mL 5% KOH (16 h at 37°C with gentle shaking) [41].

### Statistical analysis

Results were analyzed in GraphPad Prism Version 5 (Graphpad Software Inc.) and are expressed as mean ± SEM. Test for normality was performed by Kolmogorov-Smirnov, and then one-way ANOVA analysis of variance, followed by Dunnett's or Kruskall Wallis comparison test, were performed to determine any statistical differences between treated groups and untreated controls. The data were considered significant if *P*-value was <0.05.

## Results

### *In vitro* schistosomicidal activity of APLs

**Figure 2:**
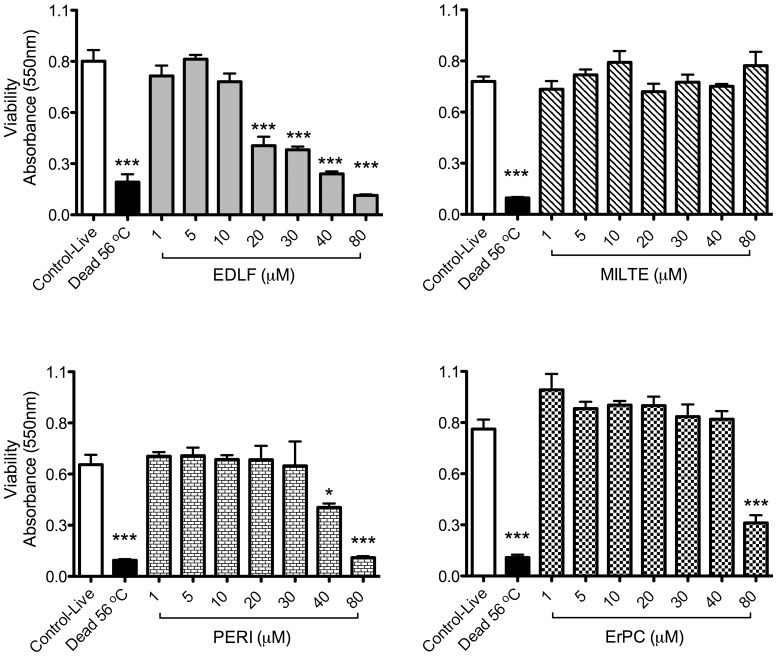
*In vitro* effects of APLs on the viability of *S. mansoni* adult worms measured by MTT assay. Couples of adult worms were treated with edelfosine (EDLF), miltefosine (MILTE), perifosine (PERI) or erucylphosphocholine (ErPC) at the indicated concentrations for 168 h. Data are means ± SEM of three separate experiments. Asterisks represent statistical significance with respect to control-live group. (*) P<0.05; (***) P<0.001.

First we analyzed the schistosomicidal activity of the four most clinically relevant APLs, namely edelfosine, perifosine, miltefosine and erucylphosphocholine, used at the range of 1–80 µM, against adult worms. By testing parasite viability using the MTT assay after 168 h incubation, we found that edelfosine was the most active APL in killing *S. mansoni* adult worms, being effective at 20 µM, whereas miltefosine was inactive even at the highest concentration used (80 µM) (Figure 2). Perifosine decreased parasite viability at 40 µM and erucylphosphocholine at 80 µM (Figure 2). The differential schistosomicidal activity of the above APLs was further supported by the microscopic observation of *S. mansoni* adult worms incubated as above with the four APLs at a concentration range of 1–80 µM to examine separation of coupled pairs, mortality, decrease in motor activity, and tegumental alterations as shown in Table 1 and Tables S1–S3. Again, edelfosine was the most potent APL in promoting parasite death (50% at 10 µM and 100% at 20 µM) in *S. mansoni* adult forms after 168 h treatment (Table 1). Incubations with 1 and 5 µM edelfosine resulted in effects on motor activity and tegumental alterations (Table 1), indicating that *S. mansoni* adult forms were very sensitive to edelfosine treatment. Perifosine also showed significant schistosomicidal activity, but higher drug concentrations were required to achieve similar effects to those exerted by edelfosine (Table 1; Tables S1–S3). In contrast, erucylphosphocholine (erucyl-PC) and miltefosine required very high doses of>40 µM to elicit some effects on the parasites (Table 1). On the basis of these morphological observations, erucylphosphocholine was only able to kill parasites at 80 µM, whereas miltefosine was unable to promote parasite death even at that concentration (Table 1), thus further supporting the above MTT data.

**Table 1:** *In vitro* effects of APLs on 56-day-old *S. mansoni* worms upon 168 h of culture.

Samples	Number of worms	Number of separated worms	Number of dead worm	Motor activity reduction		Number of worms with tegumental alterations	
				Slight	Significant	Partial	Extensive
Control-Live a	8	-	-	-	-		-
Dead 56°C b	8	-	8	-	8	4	4
PZQ c	8	-	8	-	8	-	8
Edelfosine							
1 µM	8	-	-	2	-	4	-
5 µM	8	6	-	2	6	4	4
10 µM	8	8	4	4	4	2	6
20 µM	8	8	8	-	8	-	8
30 µM	8	8	8	-	8	-	8
40 µM	8	8	8	-	8	-	8
80 µM	8	8	8	-	8		8
Miltefosine							
1 µM	8	-	-	-	-	-	-
5 µM	8	-	-	-	-	-	-
10 µM	8	-	-	-	-	-	-
20 µM	8	-	-	-	-	-	-
30 µM	8	-	-	-	-	-	-
40 µM	8	-	-	4	-	-	-
80 µM	8	4	-	6	-	-	4
Perifosine							
1 µM	8	2	-	-		-	-
5 µM	8	-	-	-		-	-
10 µM	8	2	-	4	-	6	-
20 µM	8	8	2	6	2	6	2
30 µM	8	8	2	-	8	2	8
40 µM	8	8	6	2	6	-	8
80 µM	8	8	8	-	8	-	8
Erucylphosphocholine							
1 µM	8	-	-	-	-	-	
5 µM	8	-	-	-	-	-	
10 µM	8	-	-	-	-	-	
20 µM	8	-	-	-	-	-	
30 µM	8	4	-	-	-	-	
40 µM	8	8	-	2	6	2	2
80 µM	8	8	8	-	8	-	8

a RPMI 1640.

b Control – positive.

c Tested at concentration of 10 µM.

### Dose- and time-dependent effects of edelfosine on *S. mansoni* adult worms

**Figure 3:**
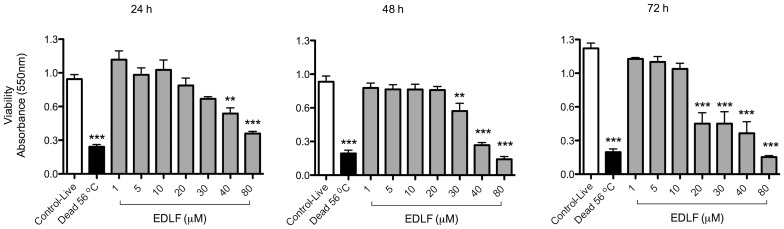
*In vitro* effects of edelfosine (EDLF) on the viability of *S. mansoni* adult worms measured by MTT assay. Couples of adult worms were treated with EDLF at the indicated concentrations for 24 h, 48 h and 74 h. Data are means ± SEM of three separate experiments. Asterisks represent statistical significance with respect to control-live group. (**) P<0.01; (***) P<0.001.

**Figure 4:**
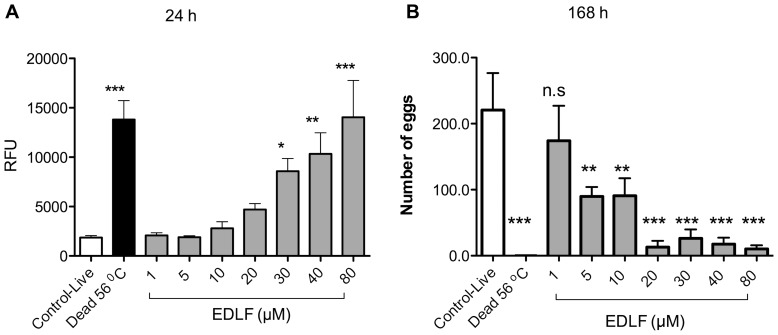
*In vitro* effects of edelfosine (EDLF) on the viability of *S. mansoni* adult worms measured by propidium iodide (PI) permeabilization and *in vitro* effects of edelfosine on egg production. Couples of adult worms were treated with EDLF at the indicated concentrations for 24 h (A) or 168 h (B), and then analyzed for membrane permeabilization using PI staining (A) and for the number of laid eggs, monitored using an inverted microscope (B). RFU, relative fluorescence units. Data are means ± SEM of three separate experiments. Asterisks represent statistical significance with respect to control-live group. (**) P<0.01; (***) P<0.001.

Because edelfosine was the most potent APL against *S. mansoni* adults, we further analyzed the effects of this drug on these worms. A time-course analysis of the effect of different concentrations of edelfosine showed that this drug could kill *S. mansoni* adult worms when incubated at 20 µM for 72 h (Figure 3) and at 30 µM for 48 h incubation (Figure 3). This is of interest as this concentration range was similar to that found as steady-state concentration of edelfosine in plasma (10–20 µM) in animal model *in vivo* assays [21], [22], [42]. The induction of structural damage and death in *S. mansoni* adult parasites by edelfosine was further supported by time-lapse videomicroscopy (Video S1), showing an increased intestinal lumen and oral as well as ventral sucker paralysis. In addition, membrane permeabilization was detected by incorporation of propidium iodide (PI) into dead parasites (Figure 4A). Parasite motility was also observed and measured under an inverted light microscope, as lack of motility represents the death of the parasite. We found that low concentrations of edelfosine (5–10 µM) were enough to promote structural changes in the parasite, both in motor activity reduction and in the tegument, as well as to split the pairs of coupled adult worms into individual female and male worms (Table 1 and Video S1), the latter being more affected than the former following incubation with 10 µM edelfosine.

### Effect of edelfosine on the egg production of *S. mansoni* adult worms

*Schistosome* eggs are considered a major cause of liver injury and illness. About half of the laid eggs lodge in the liver sinusoids, where they are trapped acting as granulomatogenic triggers, granuloma formation being strictly dependent on viable mature eggs [43]. In order to evaluate the action of edelfosine on egg production by adult worms of *S. mansoni*, edelfosine was incubated at different concentrations for 168 h. As shown in Fig. 4B, edelfosine (5–80 µM) treatment showed a significant decrease in the number of eggs after 168 h of incubation. The effect of edelfosine on the separation of the coupled adult worms could be related to this decrease on egg production.

### Distribution of fluorescent edelfosine analog in pairs of adult *S. mansoni* worms

**Figure 5:**
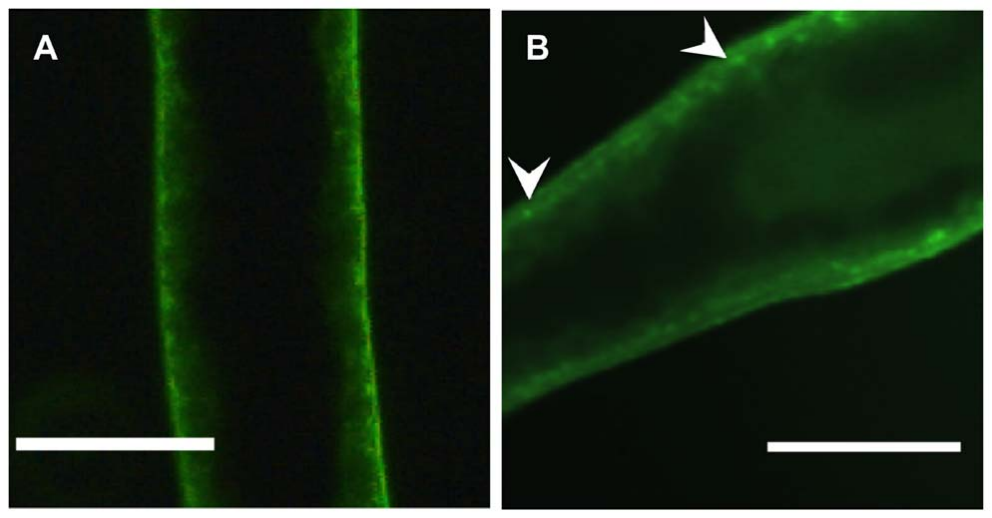
Distribution of the fluorescent edelfosine analog in *S. mansoni* adult female worms. A) Control of autofluorescence in a female cultured in RPMI-1640 only. B) Female incubated with the fluorescent edelfosine analog. Arrows show a slightly increased staining at the tegument. Scale bar, 0.2 mm.

The major interface and physical barrier between the schistosome and its external environment is through the tegument, consisting of a syncytium enclosed by a double outer tegumental membrane made up of two apposed lipid bilayers, which coats the worm and is a prime site of intimate host-parasite interaction. It performs vital functions and is of crucial importance for modulation of the host response and parasite survival [28]. By using the fluorescent edelfosine analog 1-*O*-[11′-(6″-ethyl-1″,3″,5″,7″-tetramethyl-4″,4″-difluoro-4″-bora-3a″,4a″-diaza-s-indacen-2″-yl)undecyl]-2-*O*-methyl-*rac*-glycero-3-phosphocholine, which has been previously used as a reliable analog of edelfosine to analyze its subcellular localization in single cells [37], [38], we found that adult *S. mansoni* worms took up the fluorescent analog, showing a rather diffuse staining in the whole worm, although it appeared to be most concentrated at the tegument (Figure 5). Thus, these data indicate that edelfosine is incorporated at the adult worm with a major tegumental location.

### Edelfosine induces apoptosis-like death in *S. mansoni* adult worms and changes at the tegument

**Figure 6:**
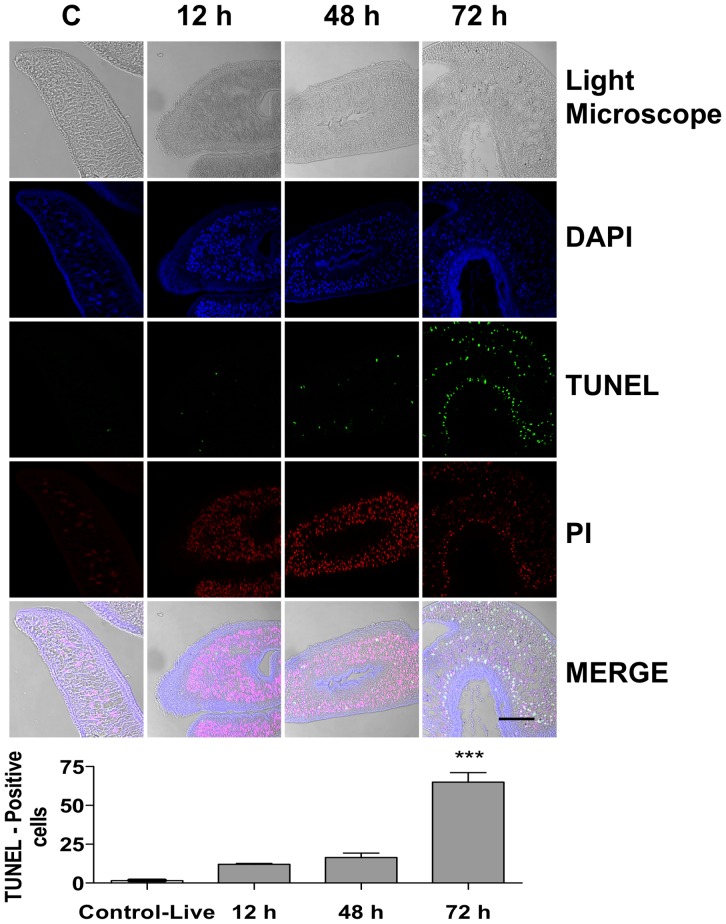
Edelfosine-induced apoptosis-like death as assessed by TUNEL assay. Adult worms were untreated (Control) or treated with 20 µM edelfosine (EDLF) for 12 h, 48 h and 72 h. Worms were analyzed by fluorescence microscopy for propidium iodide (PI) staining and TUNEL assay, as well as for DAPI staining (nuclei) and light microscopy morphology. Merging of PI and TUNEL panels (Merge) shows the apoptotic nuclei in yellow. Data shown are representative of four independent experiments. Histograms indicate the percentage of TUNEL-positive cells, as an estimate of cells undergoing apoptosis. For each experiment, at least 100 cells were analyzed. Data are means ± SEM of three separate experiments. Asterisks represent statistical significance with respect to control-live group. (***) P<0.001. Scale bar, 100 µm.

**Figure 7:**
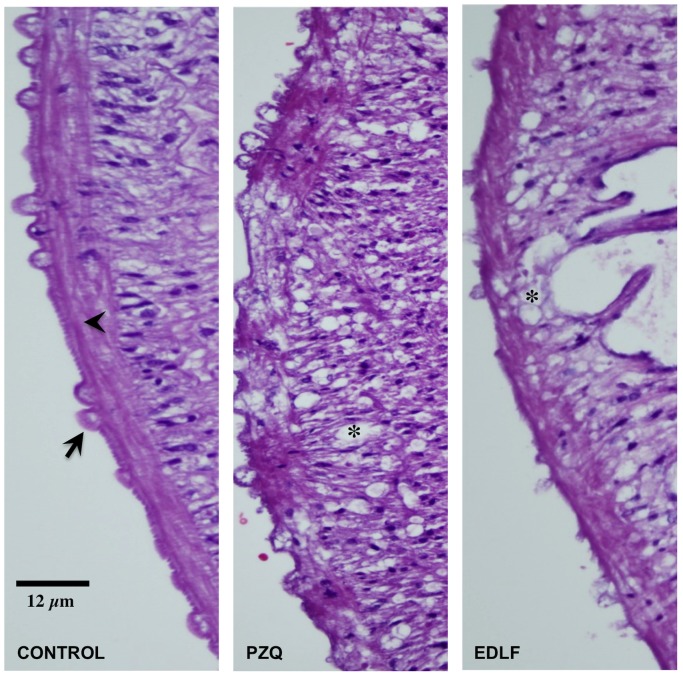
Morphological changes in *S. mansoni* adult worms following praziquantel or edelfosine treatments. H&E stained sections of freshly recovered male parasites that were untreated (Control) or treated with 10 µM praziquantel (PZQ) or 20 µM edelfosine (EDLF) for 2 days. Tubercle collapse (arrow), damages of the tegument surface, vacuolization of the subtegumental cells (asterisk) and destruction of muscle layers (arrowhead) were observed after treatment with PZQ or EDLF.

Induction of apoptotic cell death is one of the main mechanisms by which edelfosine exerts its antitumor action [20], [24], [25]. On these grounds, we analyzed whether edelfosine could promote apoptosis in *S. mansoni* adult worms, by using the terminal deoxynucleotidyl transferase-mediated dUTP nick-end labeling (TUNEL) technique for detecting DNA damage. Labeling of the 3′-OH ends of DNA, generated by DNA fragmentation, through incorporation of fluorescein-12-dUTP allowed visualization of apoptotic cells as green fluorescent cells. Worms were permeabilized and stained with 4′,6-diamidino-2-phenylindole (DAPI) and propidium iodide (PI) to visualize all nuclei from both nonapoptotic and apoptotic cells in blue and red, while TUNEL-positive cells were stained yellow in the row of the merged images and non apoptotic cells as pink fluorescent cells. We found a potent apoptosis-like response in edelfosine-treated *S. mansoni* adult worms, this apoptotic-like response being particularly abundant in cells close to the tegument (Figure 6). This edelfosine-induced apoptosis-like response increased with the incubation time (Figure 6). In addition, edelfosine treatment led to severe lesions of the tegument and subtegument tissues in both male and female worms, such as: *i)* tubercle collapse; *ii)* exfoliation and erosion of the tegument surface together with exposure and vacuolization of the subtegumental cells in the perinuclear cytoplasm of syncytium and epithelium; *iii)* extensive destruction of muscle layers (Figure 7 and Video S1).

### *In vivo* schistosomicidal activity of edelfosine in mice infected by *S. mansoni*

**Figure 8:**
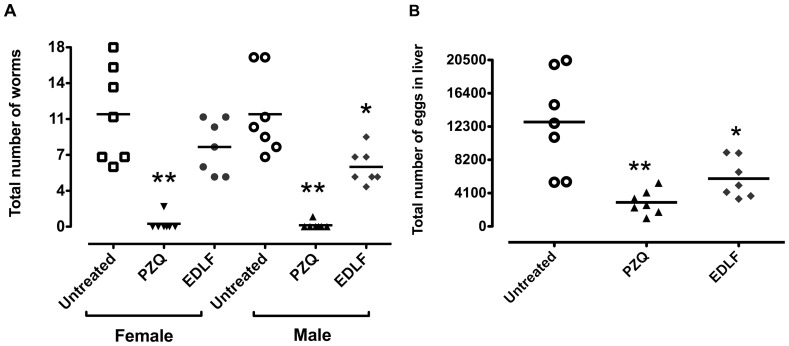
Effect on adult worm and egg burdens after treatment of *S. mansoni*-infected mice with praziquantel (PZQ) or edelfosine (EDLF). (A) male and female worm burdens. (B) egg burden in liver. Infected mice were treated with 500 mg/kg (x2) PZQ or 45 mg/kg (x10) EDLF. Each point represents data from an individual mouse. Horizontal bars indicate average values. (*) P<0.05. (**) P<0.01.

Daily oral administration of 15 or 30 mg/kg edelfosine was well tolerated by CD1 mice, 45 mg/kg being the maximum tolerated dose, following toxicity analyses, where animals were monitored for body weight loss or any appreciable side effect, including changes in strength and general condition. We found that oral treatment of mice infected with *S. mansoni* led to a reduction in the male (46.84 %) and female (29.1 %) worm burdens (Figure 8A), the decrease in male worm burden being statistically significant. PZQ treatment led to a practically complete depletion of adult worms (Figure 8A), so praziquantel treatment was more potent than that of edelfosine. Interestingly, edelfosine treatment dramatically decreased the total number of eggs recovered in livers (54.2% reduction), this inhibition in egg production being statistically significant as was the corresponding reduction (77%) exerted by praziquantel (Figure 8B).

## Discussion

Our results show the first evidence for the *in vitro* and *in vivo* schistosomicidal activity of the ether phospholipid edelfosine. This ether lipid is considered as the prototype of APLs, a family of compounds that act as promising drugs for diverse biomedical applications, including the treatment of parasitic diseases [19], [44]. Two previous reports have shown *in vivo* [28] and *in vitro* [45]. schistosomicidal activity of the APL miltefosine. However, here we found that edelfosine showed a more potent schistosomicidal activity, and APLs ranked edelfosine> perifosine> erucylphosphocholine> miltefosine for their *in vitro* activity against *S. mansoni* adult worms. Edelfosine shows also *in vivo* schistosomicidal activity, and although its activity is not as potent as that of praziquantel, the current drug of choice for schistosomiasis treatment, our data indicate that oral treatment with edelfosine decreases significantly the egg burden in liver, a key event in schistosomiasis. Our present data suggest that edelfosine is incorporated mainly in the worm tegument, promoting tegumental alterations, membrane permeabilization, motility impairment, blockade of male-female pairing and additional structural changes, which eventually lead to parasite death. In addition, we showed the induction of apoptosis-like cell death in cells apposed to the tegument, as visualized by TUNEL assays.

The integrity and function of the surface tegumental membrane are critical to the survival and proliferation of *Schistosoma*. The tegument, a syncytium covered by two lipid bilayers that is a unique structure to all trematodes [46]–[49]. In fact the two lipid bilayer structure pertains only to trematodes that live within blood vessels [50], plays vital roles like evasion of the immune system, acquisition of nutrients, excretion of catabolic products, targeted drug absorption and other physiological processes [47], [49], [51]–[54]. Most of the drugs active against schistosomiasis damage the worm tegument, including praziquantel [55], oxamniquine [56], artemether [57], mefloquine [34], [58], atorvastatin alone or in combination with medroxyprogesterone acetate [59], and thioxo-imidazoline compounds [60]. Scanning electron microscopy studies have recently shown that miltefosine induces severe tegumental damage in adult schistosomes [28], [45], [61]. In this regard, our present data on the tegumental damage elicited by edelfosine agree with those previously reported with miltefosine [28], [45], thus suggesting that APLs could be promising drugs for schistosomiasis. Furthermore, miltefosine has been reported to have *in vitro* ovicidal, larvicidal as well as lethal activity on adult worms of *S. mansoni* and *S. haematobium*, and displays molluscicidal activity on their snail hosts [61]. Miltefosine is approved for use in humans against leishmaniasis [27], [62], and pharmacokinetic as well as different studies on animal models have shown the relative lack of significant toxicity of edelfosine when used at pharmacologically relevant doses [21], [22], [38], [42], [63]. On these grounds, APLs might be promising and rather safe drugs for the treatment of schistosomiasis.

Since the 1970s the treatment of schistosomiasis has relied on a single drug, praziquantel, [8], [64], [65], that is considered as the gold standard and the drug of choice, having been successfully used over the past 35 years to control schistosomiasis in many countries. This exclusive dependency on praziquantel is alarming, raising concerns about the reliance on a single drug to treat over 200 million people that might lead to the potential appearance of massive resistance to the drug [10], [11], [16], [65]–[68]. As a result, identification of alternative schistosomiasis chemotherapies is a high priority issue.

The results shown here indicate that edelfosine significantly decreases egg burden *in vitro* and *in vivo.* This effect could be due, at least in part, to the separation of male and female schistosomes parasites induced by edelfosine, since female schistosomes produce eggs only when they are in intimate association with a male [69]. Edelfosine kills mainly adult male worms through an apoptosis-like cell death mechanism. In this regard, it is worth mentioning that edelfosine is a proapoptotic agent in cancer cells [20], [24], [25], and it seems to promote an apoptosis-like process in *Leishmania* parasites [26]. The likely involvement of different mechanisms of action for the schistomicidal activities of praziquantel and edelfosine might suggest that the combination of these two drugs could promote a synergistic action and/or minimize the possibility of drug resistance, as has been proposed for the combined use of praziquantel and artemisinin derivatives or mefloquine [70], [71]. Taking together the results herein reported as well as our previous studies showing the leishmanicidal activity of edelfosine, it might be envisaged that this drug could be of special interest in the treatment of patients that are co-infected with both *Leishmania* and *Schistosoma* parasites [29], [30], [72]–[74].

The studies reported here provide for the first time compelling evidence for schistomicidal activity of edelfosine, which, together with the low toxicity profile and the anti-inflammatory activity shown by this APL [21], [22], [38], [63], warrant further studies for the putative use of edelfosine as a possible treatment against schistosomiasis.

## Supporting Information

Table S1 ***In vitro*** **effects of edelfosine and perifosine on 56-day-old** ***S. mansoni*** **worms upon 24 h of culture.**
(XLSX)

Table S2 ***In vitro*** **effects of edelfosine and perifosine on 56-day-old** ***S. mansoni*** **worms upon 48 h of culture.**
(XLSX)

Table S3 ***In vitro*** **effects of edelfosine and perifosine on 56-day-old** ***S. mansoni*** **worms upon 72 h of culture.**
(XLSX)

Video S1 ***In vitro*** **effects following edelfosine treatment on** ***S. mansoni*** **adult worms.** Untreated control group (Live) shows no alteration in morphology and was able to produce eggs. Dead worms following heating at 56°C or treatment with 10 µM praziquantel (PZQ) (positive controls) showed no motility and became nontranslucent. Worms treated with the indicated concentrations of edelfosine (EDLF) for 48 h, 72 h and 168 h showed toxic actions when used ≥ 10 µM. In the video one arrow shows severely dilated gut of a female worm and two arrows show extensive damage on the tegument of a male worm following treatment with 30 µM of EDLF for 48 h. After the 4:30 min, another arrow shows extensive peeling in the tegument of a female worm treated with 10 µM EDLF for 168 h.
(MOV)
