# Correction: *In Vitro* and *In Vivo* Anti-Schistosomal Activity of the Alkylphospholipid Analog Edelfosine

**DOI:** 10.1371/journal.pone.0123149

**Published:** 2015-04-02

**Authors:** The PLOS ONE Staff

The fourth author’s name is incorrect. The correct name is: El Habib Dakir. The correct citation is: Yepes E, Varela-M RE, López-Abán J, Dakir EH, Mollinedo F, Muro A. (2014) *In Vitro* and *In Vivo* Anti-Schistosomal Activity of the Alkylphospholipid Analog Edelfosine. PLoS ONE 9(10): e109431. doi:10.1371/journal.pone.0109431
